# Editorial: Methods and advances in induced pluripotent stem cells—ophthalmology

**DOI:** 10.3389/fcell.2023.1298956

**Published:** 2023-10-16

**Authors:** Lyle Armstrong, Majlinda Lako

**Affiliations:** Biosciences Institute, Newcastle University, Newcastle upon Tyne, United Kingdom

**Keywords:** retina, retinal ganglion cells, photoreceptors, retinal organoids, eye development

Frontiers in Cell and Developmental Biology presents an exciting series of articles encompassing significant developments in the application of Pluripotent Stem Cells (PSCs) to the treatment of ophthalmological diseases. Diseases affecting the retina and its associated innervation present significant societal challenges since treatments are frequently limited to symptom management and slowing the progression of the disease rather than affecting a complete cure. Conditions such as Age-Related Macular Degeneration (AMD) characterised by declining functions of both the retinal pigment epithelium and neural retina, and Glaucoma which involves dysfunction of retinal ganglion cells and the optic nerve, currently affect approximately 280 million individuals worldwide. Without the means to repair the damage to retinal structures, the impact of these diseases will worsen as the numbers of older individuals in the global population increases; thus the costs of disease management are likely to rise significantly. In view of these sobering observations, the number research efforts aimed at increasing our understanding of retinal degeneration and developing methods to ameliorate the decline are encouraging.

AMD is recognised in the early and intermediate stages by the appearance of sub-retinal deposits of lipids and proteinaceous material called “drusen.” Initially, these deposits have limited effect on visual acuity but approximately 15% of affected individuals progress to develop either “wet” or ‘dry AMD (Chichagova et al., 2018). The former is characterised by aberrant choroidal blood vessel growth through the RPE affecting the function of the overlying neurosensory retina by vascular leak, haemorrhage and fibrosis with subsequent outer retinal degeneration (Hobbs and Pierce, 2022), whilst dry AMD is notable for the degeneration of retinal pigment epithelial (RPE) cells and the adjacent photoreceptors. This condition can progress to geographic atrophy and/or wet AMD but there are few options for treating these forms of AMD since the affected regions of RPE or photoreceptors are often destroyed. Replacing the damaged cells by transplanting their healthy counterparts is an attractive prospect although the likelihood of success will depend on the degree of retinal damage that has occurred before the patient presents for treatment. For example, if the neural retina has suffered significant degradation leading to photoreceptor loss, replacement of these cells would be required; however if the connectivity of the neural retina is intact, replacement of the RPE alone may be sufficient. In this respect the paper by Rohowetz and Koulen in this Research Topic is exciting since it provides a highly informative review of the current state of the art in RPE transplantation. A key point arising from this publication is that the safety of RPE transplant seems to be promising and that adverse events arising during the procedure are more likely related to surgery induced damage or to the need for immunosuppression which indicates the need for more precise HLA matching; however, an alternative strategy to eliminate the need for immunosuppression might be to generate RPE cells from “hypoimmunogenic” iPSC lines in which the assembly of the MHC-I major histocompatibility complex at the cell surface is inhibited by homozygous knockout of the gene encoding β-2-microglobulin (Deuse et al., 2019; Zhao et al., 2020; Chen et al., 2023).

Glaucoma is the second leading cause of blindness worldwide (The International Bank for Reconstruction and Development/the World Bank, 1993) and the definition of this is a group of ocular diseases that cause progressive changes in the optic nerve head leading to visual field loss (American Academy of Ophthalmology, 1996). Despite considerable historical focus on intra-ocular pressure as a contributing factor to the development of the disease, Glaucoma is primarily a neurodegenerative disease arising from progressive retinal ganglion cell (RGC) loss. RGCs are the main output neurons that rely on signals generated by the photoreceptors to the brain and there are no current therapies that can replace their loss so many current research efforts are aimed at enhancing RGC survival. Once again, transplant of healthy RGCs into the retinas of glaucoma patients to recover neural signalling is an attractive concept especially since early efforts to transplant RGCs into murine glaucoma models have shown encouraging results; however human trials are still several years away (Oswald et al., 2021). As one of the earliest types of cells to arise during embryogenesis, RGCs are the focus of many research efforts (Ohlemacher et al., 2015; Chavali et al., 2020) so it is encouraging to see the advances made by Subramani et al. as published as part of this Research Topic. This group addressed the problems of variability in the generation of RGCs by using a monolayer based protocol for differentiation of pluripotent stem cells which in its early stages, resembles techniques needed for generation of many neural cell types but is later modified to drive the cells into a retinal progenitor phenotype. Expansion and banking of such retinal progenitor cells is the key to delivering large numbers of RGCs since the group’s protocol subsequently transforms the progenitors into RGCs by stepwise administration of growth factors. Moreover, RGCS produced in this manner integrate into the retina of host mice and were able to initiate axon growth.

The remaining two papers in this series add to our understanding of pluripotent stem cell derived retinal organoid development. This is an important enquiry since many of the cell types generated in 3D retinal organoids show more advanced maturity than those generated by 2D differentiation protocols (with the exception of Subramani et al. approach to making RGCs or course). Of particular value is the light responsiveness of organoid photoreceptors (Zhong et al., 2014; Hallam et al., 2018) which underlines the utility of retinal organoids as models of retinal disease. Existing retinal disease models are inadequate for studying the developmental origins of retinal diseases during embryonic development so the derivation of retinal organoids from mutation carrying or patient specific induced pluripotent stem cells allows us to recapitulate *in vivo* retinogenesis and restate disease phenotypes seen in patients (Watson and Lako, 2023). Naturally, the suitability of retinal organoids to these tasks is developing constantly so the contribution made by Völkner et al. to this series is valuable because it further optimises the process of retinal organoid generation which will be of considerable use to other groups in this field. The last publication in this series published by Wahlin et al. explores early retinal development by introducing markers of eye field development that may be used to distinguish those cells that have committed to a retinal development fate from those that may be forming cells that contribute to other regions of the CNS.

Overall, we have enjoyed reading these incisive articles and we hope that the reader will draw the same level of inspiration and enjoyment from this special series highlighting the great contributions of pluripotent stem cells to the understanding and potential treatment of eye diseases.
